# The Fluid Dynamical Performance of the Carpentier-Edwards PERIMOUNT Magna Ease Prosthesis

**DOI:** 10.1155/2018/5429594

**Published:** 2018-01-10

**Authors:** Philipp Marx, Wojciech Kowalczyk, Aydin Demircioglu, Gary Neil Brault, Hermann Wendt, Sharaf-Eldin Shehada, Konstantinos Tsagakis, Mohamed El Gabry, Heinz Jakob, Daniel Wendt

**Affiliations:** ^1^Department of Thoracic and Cardiovascular Surgery, West-German Heart and Vascular Center Essen, University Hospital Essen, Essen, Germany; ^2^Chair of Mechanics and Robotics, University Duisburg-Essen, Campus Duisburg, Lotharstraße 1, 47057 Duisburg, Germany; ^3^Institute for Diagnostic and Interventional Radiology and Neuroradiology, University Hospital Essen, Essen, Germany

## Abstract

The aim of the present in vitro study was the evaluation of the fluid dynamical performance of the Carpentier-Edwards PERIMOUNT Magna Ease depending on the prosthetic size (21, 23, and 25 mm) and the cardiac output (3.6–6.4 L/min). A self-constructed flow channel in combination with particle image velocimetry (PIV) enabled precise results with high reproducibility, focus on maximal and local peek velocities, strain, and velocity gradients. These flow parameters allow insights into the generation of forces that act on blood cells and the aortic wall. The results showed that the 21 and 23 mm valves have a quite similar performance. Maximal velocities were 3.03 ± 0.1 and 2.87 ± 0.13 m/s; maximal strain *E*_*xx*_, 913.81 ± 173.25 and 896.15 ± 88.16 1/s; maximal velocity gradient *E*_*yx*_, 1203.14 ± 221.84 1/s and 1200.81 ± 61.83 1/s. The 25 mm size revealed significantly lower values: maximal velocity, 2.47 ± 0.15 m/s; maximal strain *E*_*xx*_, 592.98 ± 155.80 1/s; maximal velocity gradient *E*_*yx*_, 823.71 ± 38.64 1/s. In summary, the 25 mm Magna Ease was able to create a wider, more homogenous flow with lower peak velocities especially for higher flow rates. Despite the wider flow, the velocity values close to the aortic walls did not exceed the level of the smaller valves.

## 1. Introduction

The incidence of heart valve disease is constantly growing in number [1, 2]. In particular, aortic stenosis is meanwhile the most common heart valve disease owing to an increased number of elderly patients. Usually, reasonable reconstruction of stenotic aortic valves is not feasible, and therefore aortic valve replacement has become the method of choice.

At a first glance, construction of valve prostheses, from a technical point of view, appears to be simple. Based on several previously designed valves, concepts are known and were widely applied. However, the construction of a hemodynamic perfect heart valve is highly sophisticated. Not only do design aspects have to be taken into account, but also blood compatibility, hemodynamics, and durability were of utmost importance. During the last decades several companies and researchers tried to develop valve prostheses that meet both biological and technical demands. By doing so, valve designs got more complex and were composed of a large number of varying materials, such as leaflets made from heterologous pericardium, and different metallic alloys were used for the inner armature of the stent.

The Carpentier-Edwards PERIMOUNT pericardial aortic bioprosthesis represents a first-generation biological heart valve, which has been implemented into the market in 1981 and is therefore one of the most well-studied bioprostheses.

Several recently published studies verified that the PERIMOUNT aortic valve showed good durability and only a low number of structural valve deteriorations (SVD) even in younger patients who had a higher risk of early degeneration of their valve [3–7].

The PERIMOUNT Magna Ease which is a further development of the PERIMOUNT Magna bioprosthesis belongs to the latest generation of aortic valve prostheses. Coming with several enhancements like especially improved hemodynamic performance, it led to a reduction in the incidence of patient-prosthesis mismatch [8–11].

In the present work, the aim was to evaluate the flow parameters of the PERIMOUNT Magna Ease in order to gain a better understanding of how the valve size and the different flow pattern influence the fluid dynamical performance of the Magna Ease.

## 2. Methods

### 2.1. Bioprostheses

The Carpentier-Edwards PERIMOUNT Magna Ease Bioprosthesis (PME) (Edwards Lifesciences, Irvine, CA, USA) is a pericardial aortic bioprosthesis with a supra-annular design. The three bovine pericardial leaflets have been treated with the ThermaFix™ process, an anticalcification technology that extracts the molecules of unstable glutaraldehyde and phospholipids, which are considered to favor calcification processes in the long term. The mechanical stability of the valve is ensured through a stent made of a flexible cobalt-chromium alloy. Compared to the Magna valve the Magna Ease was designed with a lower profile for an easier implantation and an improved coronary ostia clearance. The valve is available in 6 sizes of 19, 21, 23, 25, 27, and 29 mm.

### 2.2. Test Setup

The test setup consisted of two modules, a flow channel and a PIV (particle image velocimetry) system. The flow channel has already been described in previous studies [12–14]. The present flow channel was updated by a more physiological aortic model made of transparent silicon instead of PMMA (polymethylmethacrylate) and a modified simulation of peripheral resistance. The shape of the aortic model was representing not only the ascending part, but also the aortic arch with its branches and the descending part of the aorta. The largest inner diameter of the aorta was at the level of the sinus (34 mm) and was reduced to 27 mm diameter 30 mm above the annulus. Therefore, it was possible to test also bigger prostheses. Due to the applied PIV technology the whole flow chamber had to be transparent and should allow an ideal view on the flow inside of the aorta.

In the present study we applied typical physiological flow conditions covering a stroke volume ranging from 60 to 80 mL and a heart rate ranging from 60 to 80 beats per minute (bpm) (cardiac output of 3.6–6.4 L/min). During all experiments there was a constant aortic pressure of 120/80 mmHg. The pressures were monitored by a pressure catheter connected to the RadiAnalyzer (St. Jude Medical, Saint Paul, MN, USA). The flow was created by a membrane pump (Sigma 3Ca, ProMinent Dosiertechnik GmbH, Heidelberg, Germany). The system was filled with water at room temperature mixed with fluorescent seeding particles (diameter of 20–50 *μ*m) acting as the test fluid.

At the beginning of each experiment the calibration of all flow parameters was checked and the measurements were not started before the system was running stable for at least 10 minutes.

### 2.3. Image Analysis

For flow-analysis, a two-dimensional PIV system of the company LaVision GmbH (Göttingen, Germany) was used. This system consisted of a laser (double-pulsed Nd:YAG laser, wave-length 532 nm, maximum output 400 mJ), a high-speed camera (ImagerProHS500), and a computer system. The camera was adjusted to take double-framed pictures of the flow field with a frequency of 100 Hz and a time interval (dt) of 900 *μ*s between both frames. The comparison of these doubled-framed pictures with the illuminated seeding particles allowed precise measurements of the fluid dynamical behavior of the test fluid and the calculation of several parameters. For all software applications the DaVis 8.2.3 software (LaVision, Göttingen, Germany) was used.

Before starting, the laser and the camera had to be adjusted and calibrated. Possible optical distortions were removed or mathematically corrected during the calibration process.

The captured data was stored on a hard disk for later postprocessing.

**Figure 1:**
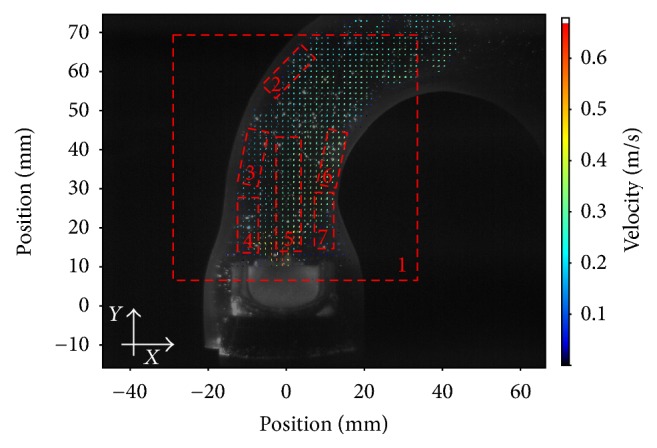
Overview of the regions of interest.

In order to allow more precise and differentiated evaluation of the flow parameters, the flow field was subdivided into seven regions of interest (ROIs) (see Figure 1). For a better orientation an *xy*-plane was introduced. The first region of interest, ROI 1, covered the whole flow field. ROI 5 was placed exactly in the middle of the ascending part, which incorporated the area of the central orifice jet. ROI 2, 3, 4, 6, and 7 covered flow areas that were very close to the aortic wall.

During evaluation five flow parameters were examined: (1) the maximal velocities that occurred in each cardiac cycle, where we focused on each single vector with the highest velocities measured; in contrast to other studies, obtained data were not based on average values; (2) the normal strain in *x*-direction *E*_*xx*_ = ∂*V*_*x*_/∂*x*; (3) the normal strain in *y*-direction *E*_*yy*_ = ∂*V*_*y*_/∂*y*; (4) the velocity gradient *E*_*xy*_ = ∂*V*_*x*_/∂*y* that describes the change of *V*_*x*_ along the *y*-axis direction and represented a horizontal shear; (5) the velocity gradient *E*_*yx*_ = ∂*V*_*y*_/∂*x* representing the vertical shear.

The strain represents the compression or expansion of the fluid depending on the values being either positive or negative.

The combined examination of these parameters enabled good estimation of the maximal forces acting on the single blood cells, the leaflets of the valves, and the aortic wall. To simplify, we only used the positive values for the statistics. The negative values were on the same level and identical.

### 2.4. Statistics

The preprocessed data of the measurements were performed by the software DaVis 8.2.3. A two-way ANOVA was performed evaluating the maximum velocity depending on valve size and heart rate. Outliers were detected by using Cooks Distance and were subsequently removed. The normality assumption of the ANOVA was tested by a Shapiro-Wilk normality test, whereas the Levene test was applied to verify the homogeneity of variance of the data. In case the two assumptions were not met by the data, a nonparametric Scheirer-Ray-Hare test, which does not depend on both assumptions, was conducted and its results were compared to the two-way ANOVA test. A post hoc pairwise *t*-test with adjustment for multiple testing was applied subsequently to find significant differences between the groups. The statistical tests were performed with the R programming environment using the car and the rcompanion package. Differences were considered to be significant for *P* values < 0.05.

## 3. Results

**Figure 2:**
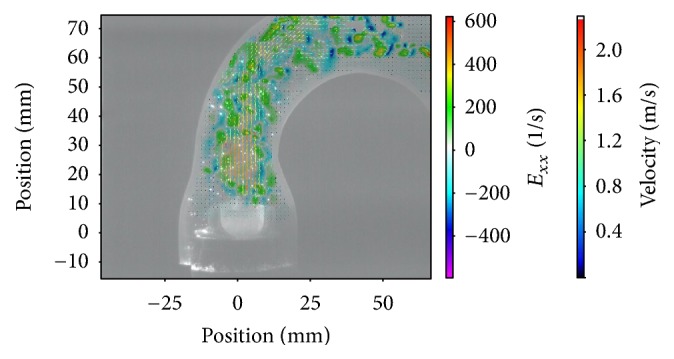
Magna Ease 21 mm *E*_*xx*_ flow 70 bpm.

**Figure 3:**
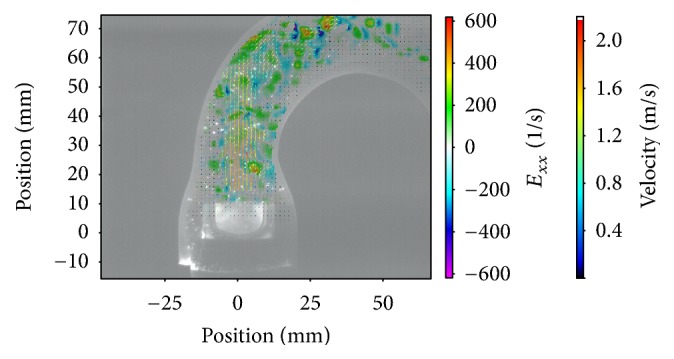
Magna Ease 23 mm *E*_*xx*_ flow 70 bpm.

**Figure 4:**
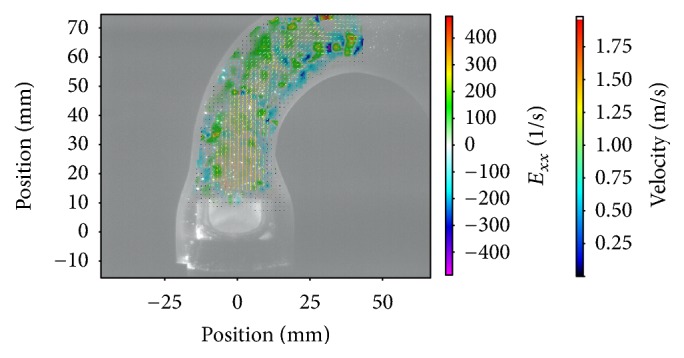
Magna Ease 25 mm, *E*_*xx*_, flow 70 bpm.

The results were based on the evaluation of 380 cardiac cycles. More than 68000 double-framed pictures were taken in total. With the application of a complex postprocessing procedure it was possible to visualize a precise and detailed vector field for each image (e.g., Figures 2–4).

In general the flow of all valves showed the profile of a typical triangular velocity profile with recirculating regions on the level of the sinus cavity and further vortices near the aortic wall.

### 3.1. Velocities

**Figure 5:**
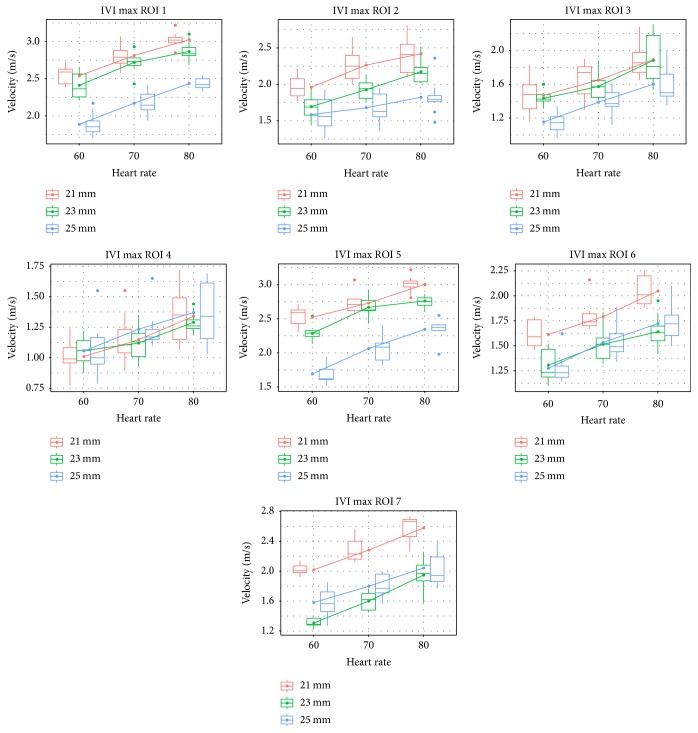
Maximal velocities ROI 1–7.

The plots (Figure 5) revealed the existence of some outliers, as measuring the maximum velocity or gradient is not robust. Therefore, outliers were detected by computing the Cook's distance for all points, and those values having a distance more than four times the mean distance were removed.

The highest velocities were measured in ROI 1 (Figure 5). As ROI 1 covered the whole flow field, these results could also be used as a control measurement. Computing the mean average difference (MAE) of the velocities of the subdivided ROIs showed clearly that the velocity values of ROI 1 were very similar to the central ROI 5 (MAE = 0.095 m/s), and they were much higher for the other ROIs (MAE between 0.598 and 1.350 m/s). So it can be concluded that the highest peak velocities primarily occurred in the area of the central jet.

A two-way factorial ANOVA was conducted to compare the main effects of the two factors, valve size and heart rate, and the interaction effects between those on the maximum velocity. Valve size consisted of three levels (21 mm, 23 mm, 25 mm) and heart rate included three levels (60, 70, 80 bpm). The results for all ROIs (Table 1) indicated that there were no significant interactions between both factors, mm and bpm, and for all ROIs, except for ROI 4, both factors, mm and bpm, were significant. For ROI 4 the mm factor was not significant. This can clearly be seen in the plot, where all velocities are very close to each other.

The assumptions of the ANOVA, normality of the residuals and the homogeneity of the variance across the groups, were tested by a Shapiro-Wilk test and a Levene test, respectively. The former test showed that the normality assumption could not be rejected for all ROIs, except for ROI 6 (*P* < 0.001). By detailed data evaluation of ROI 6, this result was related to outliers that were not removed by the outlier detection. However, as the data was balanced, it was suspected that the ANOVA test was not impacted by this deviation of normality. To further control for bias, a Scheirer-Hare-Ray test was conducted especially for ROI 6. This test acts a nonparametric equivalent of the two-way ANOVA that does not depend on the normality assumptions of the ANOVA and showed finally the same significance. It indicated no interaction between the two factors mm and bmp, but both factors were highly significant. The Levene test showed no significant results for any ROI; that is, the variances were homogeneous. See Tables 2 and 3 for more details.

Differences between the groups were tested by a pairwise *t*-test, where the *P* values were adjusted with the Holm method to account for multiple testing. Overall, in ROI 1, there was a significant, but not particularly large, difference between the 21 mm and the 23 mm heart valves (*P* = 0.036). The same holds true for the central area (ROI 5) and the right-sided areas, where the differences are highly significant (*P* < 0.01). On the left areas, the differences between the two valves were clearly not significant (*P* = 0.474 for ROI 3 and *P* = 0.927 for ROI 4). The differences between the 21 mm and the 25 mm heart valves were highly significant for all ROIs (*P* < 0.01) except for ROI 4 (*P* = 0.927). Finally, the differences between the 23 mm and 25 mm heart valves again were highly significant for all ROIs (*P* < 0.01) except for ROI 4 (*P* = 0.927) and ROI 6 (*P* = 0.718).

In summary, the central areas and the right areas showed a significant difference between all valves sizes, while on the left side, the differences between the valves were not that different. The 25 mm valve was superior to the 21 and 23 mm ones, which showed similar velocities.

### 3.2. Normal Strain and Velocity Gradients

The normal strain and the velocity gradients were calculated only for the complete flow field (ROI 1).

**Figure 6:**
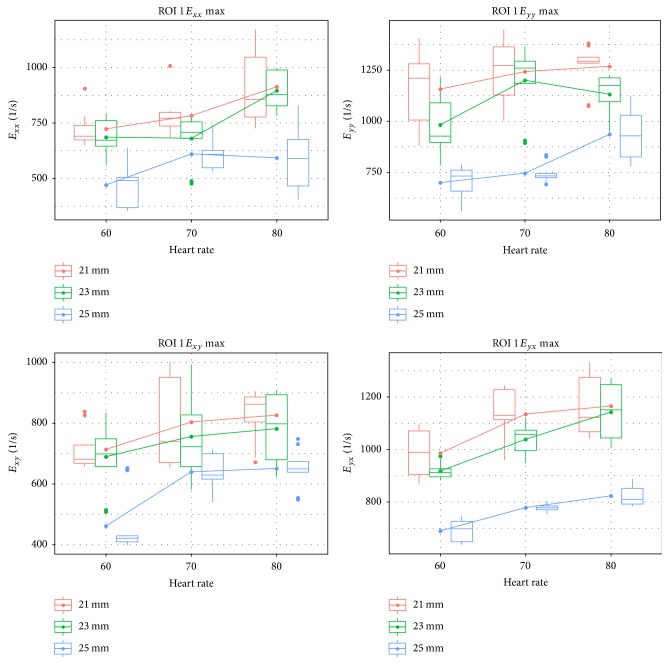
Normal strain and the velocity gradients.

The plots for the normal strain and velocity gradients (Figure 6) again revealed several outliers, which were removed by applying the Cook's method.

A two-way factorial ANOVA was conducted to compare the main effects of the two factors, valve size and heart rate, and the interaction effects between them on the maximum of the gradients. The results for the gradients indicated a significant interaction between both factors, mm and bpm, for the velocity gradients *E*_*xx*_ (*P* = 0.032) and *E*_*yy*_ (*P* = 0.046). No such interaction was visible for the normal strain gradients, *E*_*xy*_ (*P* = 0.479) and *E*_*yx*_ (*P* = 0.317). Both factors, mm and bpm, were highly significant (*P* < 0.01).

Again, the assumptions of the ANOVA, normality and variance homogeneity, were tested. For all gradients, the normality assumption could not be rejected. The Levene test showed a significant violation only for *E*_*yx*_, but not for the other gradients. Because of this, a Scheirer-Ray-Hare test, which is not sensitive to the heterogeneity of the variance, was applied to Grad *E*_*yx*_ to verify the results of the ANOVA. The results of the Scheirer-Ray-Hare test showed the same significance as the ANOVA test; that is, the interaction between the two factors was not significant (*P* = 0.952), but both factors were highly significant (*P* < 0.01).

All groups were tested for differences by a pairwise *t*-test with Holms method. The differences between the 21 mm and the 23 mm heart valves were not significant for the two gradients *E*_*xx*_ (*P* = 0.109) and *E*_*xy*_ (*P* = 0.263), but they were significant for the gradients *E*_*yx*_ and *E*_*yy*_ (both *P* < 0.01). On the other hand the differences between the 21 mm and the 25 mm were highly significant (*P* < 0.01) for all four gradients. This was also true for the differences between the 21 mm and the 25 mm heart valves (again *P* < 0.01).

In summary the results of these parameters were in line with the velocity measurements. The 21 mm and 23 mm were not clearly different for all gradients, but the 21 mm and 25 mm as well as the 23 mm and 25 mm heart valves clearly differed. Again the 25 mm valve was superior to the 21 and 23 mm valves.

Besides the statistical analyses of the measured parameters, the evaluation of the visualized vector fields was important as it provided further and more detailed information. Figures 2–4 depict the normal strain in *x*-direction *E*_*xx*_, which was especially high in the lateral peripheral areas of the central jet. The high mechanical load in this area became more obvious by evaluating the isolated velocity gradient *E*_*yx*_.

Due to the wider and more homogeneous central jet of the 25 mm valve, the velocity gradients and the strain were reduced. For this valve the velocities in general were lower and additionally the transition between the central jet and the areas with lower velocities was smoother than that for the 21 and 23 mm valves (Figures 2–4).

The comparison of the velocity gradients of the 21 mm valve with the velocity gradients of the 23 and 25 mm ones showed that the areas with high gradients (in this case negative values) of the 21 mm valve were getting very close to the aortic wall. This is strong evidence that the central orifice jet was not completely centered and it explained the high velocities of the 21 mm valve in the right-sided ROIs 6-7, while the values in ROI 3-4 were lower.

## 4. Discussion

The present study intended to continue our research work that was already dealing with the comparison of the Carpentier-Edwards PERIMOUNT Magna and the Carpentier-Edwards PERIMOUNT Magna Ease bioprostheses [12, 13].

In this study we decided to use a different strategy for the evaluation of the flow parameters. As only the highest single velocity vectors of the whole cardiac cycles were taken into account, this study was designed to evaluate more precisely the maximal velocities and maximal mechanical loads that were acting on local areas in the flow field. However, this different approach has to be considered when the results are compared with the velocity values of other investigations. Most of the present studies focused, for example, on the highest average velocities that occurred during the peak flow phase [15–17], so the values of this study were consequently slightly higher.

As one might expect the velocity values of ROI 1 showed that the valves with bigger sizes tended to have a lower velocity profile than the smaller ones. These findings reflect the continuity law which says that

(1) $$\begin{array}{c} Q=A_{1}\times v_{1}=A_{2}\times v_{2}, \end{array}$$

where *Q* is the flow rate, *A*_1_ cross-sectional area 1, *A*_2_ cross-sectional area 2, *v*_1_ flow velocity 1, and *v*_2_ flow velocity 2.

If we assume an almost round shape of the effective orifice area, the cross-sectional area can be estimated by *A* = *πr*^2^ (*r* = radius of the effective orifice area).

On the basis of this physical principle it can be expected that the size (diameter) of the same type of valve has a major influence on the velocities. Prior investigations already showed that the effective orifice area of the Magna Ease indeed increased depending on its size [15, 18].

Therefore it is remarkable that the peak velocity values were very similar in some regions (e.g., ROI 4, flow: 80 bpm) irrespective of valve size. In most ROIs and during most flow conditions especially the 21 and 23 mm valves showed only minor differences.

However, the equation of the fluid dynamical performance of both valves might be a premature conclusion. In the small right-sided ROIs 6 and 7 significant and obvious velocity differences could be observed. The increased values in these areas were important as they were very close to the aortic wall. Higher velocities or vortices in combination with higher values for strain and increased velocity gradients represent a higher biomechanical load on the aortic tissue and are assumed to have a strong impact on pathological processes [19]. Therefore, a relation between hemodynamics and atherosclerotic lesions was observed [20].

Without the extreme precise and detailed PIV measurement of this study the small differences between the 21 mm and 23 mm valve would be difficult to detect.

On the other hand, even the single peak values of the 21 mm valve were still much lower than average flows that are considered to be critical in patients suffering from aortic valve stenosis [21]. Even the highest velocities that were measured over the course of all experiments did not exceed 3 m/s and were measured only in the central jet area. Against the background of all the results, the flow profile of the 21 mm and the 23 mm valve can still be considered similar.

Nevertheless, the findings showed how precise and valuable PIV measurements can be for the testing of heart valve prostheses in general.

The evaluation of the strain parameters and the velocity gradients indicated that the superiority of the 25 mm valve was caused by a more constant, wider, and homogenous flow profile. This was particularly obvious looking at the vector fields during the peak flow phase (Figures 2–4).

As the central jets of the 21 and 23 mm valves seemed to be narrower than the one of the 25 mm valve, it might appear to be strange that in an aorta with a relatively large diameter the small 21 mm valve produced the highest velocity levels close to the aortic wall (few millimeters above the valve).

For the understanding of this phenomenon it has to be taken into account that each valve, independent of its size, was placed in the same standardized aortic model and consequently the relative geometric proportions were different. So there was a different lateral distance between the narrow central orifice jets of the small valves compared to the wider jet of the larger valves. As a consequence the height and the formation of the first vortices varied.

Even though we tried to place the valves exactly in the middle of the aortic model, it might have been possible that the vertical axis of the valves showed an angle of less than 2 degree. It is possible that the high velocities of the 21 mm valve on the right side of the aortic valve were, to some extent, caused by such a displacement. As the heart is a highly dynamic and flexible organ, it can be assumed that in vivo the velocities close to the aortic wall are varying more strongly, dependently on the placement of the prostheses and the individual anatomical circumstances in each patient.

As we used water instead of a water-saline or glycerin solution as blood similar test fluid [22] the validity of the absolute values of the collected data might be limited. Although previous studies showed that for the qualitative and relative comparison the use of water is acceptable [12, 13].

## 5. Conclusion

The present study proved that valves of the same type, but different in size, showed a similar and characteristic fluid dynamical performance in general. However, under specific circumstances the chosen valve sizes can not only lead to variations of the flow velocities (in case of a bigger valve) but also create different and individual local flow patterns. In this context the investigation of a variety of applied cardiac outputs is important for a complete evaluation of the valves as it creates a broader picture of possible flow fields. Additionally, it has to be taken into account that the flow interacts also with other anatomical conditions, such as the size of the aorta. In this study we observed only minor differences between the 21 mm and the 23 mm valves while there were significantly reduced velocities and mechanical loads for the 25 valve. For future investigations in vitro and in vivo tests should always try to include valves not only of different types but also of different sizes.

## Figures and Tables

**Table 1:** Results of the two-way factorial ANOVA.

	Valve size (mm)	Heart rate (bpm)	Interaction between valve size and heart rate (mm:bpm)
Grad *E*_*xx*_	**P < 0.001** **F(**2,79**) = 41.2**	**P < 0.001** **F(**2,79**) = 17.72**	**P = 0.032** **F(**4,79**) = 2.79**
Grad *E*_*xy*_	**P < 0.001** **F(**2,77**) = 27.24**	**P < 0.001** **F(**2,77**) = 13**	*P* = 0.479 *F*(4,77) = 0.88
Grad *E*_*yx*_	**P < 0.001** **F(**2,75**) = 145.47**	**P < 0.001** **F(**2,75**) = 39.55**	*P* = 0.317 *F*(4,75) = 1.2
Grad *E*_*yy*_	**P < 0.001** **F(**2,79**) = 77.88**	**P < 0.001** **F(**2,79**) = 11.6**	**P = 0.046** **F(**4,79**) = 2.55**
ROI 1	**P < 0.001** **F(**2,78**) = 182.26**	**P < 0.001** **F(**2,78**) = 97.19**	*P* = 0.733 *F*(4,78) = 0.5
ROI 2	**P < 0.001** **F(**2,78**) = 47.23**	**P < 0.001** **F(**2,78**) = 28.41**	*P* = 0.273 *F*(4,78) = 1.31
ROI 3	**P < 0.001** **F(**2,78**) = 20.23**	**P < 0.001** **F(**2,78**) = 38.92**	*P* = 0.929 *F*(4,78) = 0.21
ROI 4	*P* = 0.397 *F*(2,76) = 0.93	**P < 0.001** **F(**2,76**) = 19.27**	*P* = 0.867 *F*(4,76) = 0.32
ROI 5	**P < 0.001** **F(**2,76**) = 199.52**	**P < 0.001** **F(**2,76**) = 98.94**	*P* = 0.063 *F*(4,76) = 2.34
ROI 6	**P < 0.001** **F(**2,77**) = 38.3**	**P < 0.001** **F(**2,77**) = 48.15**	*P* = 0.631 *F*(4,77) = 0.65
ROI 7	**P < 0.001** **F(**2,76**) = 129.8**	**P < 0.001** **F(**2,76**) = 80.26**	*P* = 0.606 *F*(4,76) = 0.68

**Table 2:** Normality test and homogeneity of variance test.

	Grad *E*_*xx*_	Grad *E*_*xy*_	Grad *E*_*yx*_	Grad *E*_*yy*_	ROI 1	ROI 2	ROI 3	ROI 4	ROI 5	ROI 6	ROI 7
Normality test (Shapiro-Wilk)	0.156	0.155	0.587	0.064	0.176	0.248	0.363	0.072	0.361	**0.001**	0.888
Homogenity of variance test (Levene)	0.062	0.084	**<0.001**	0.067	0.544	0.723	0.142	0.050	0.871	0.991	0.101

**Table 3:** Significance of pairwise *t*-tests.

	Grad *E*_*xx*_	Grad *E*_*xy*_	Grad *E*_*yx*_	Grad *E*_*yy*_	ROI 1	ROI 2	ROI 3	ROI 4	ROI 5	ROI 6	ROI 7
Valve size 21 mm versus 23 mm	0.109	0.263	**0.043**	**0.005**	**0.036**	**<0.001**	0.474	0.927	**0.007**	**<0.001**	**<0.001**
Valve size 21 mm versus 25 mm	**<0.001**	**<0.001**	**<0.001**	**<0.001**	**<0.001**	**<0.001**	**<0.001**	0.927	**<0.001**	**<0.001**	**<0.001**
Valve size 23 mm versus 25 mm	**<0.001**	**<0.001**	**<0.001**	**<0.001**	**<0.001**	**<0.001**	**<0.001**	0.927	**<0.001**	0.718	**0.014**

## References

[1] Iung B., Baron G., Butchart E. G. (2003). A prospective survey of patients with valvular heart disease in Europe: The Euro Heart Survey on valvular heart disease. *European Heart Journal*.

[2] Vahanian A., Alfieri O., Andreotti F. (2012). Guidelines on the management of valvular heart disease of the European Society of Cardiology (ESC) and the European Association for Cardio-Thoracic Surgery (EACTS''). *European Journal of Cardio-Thoracic Surgery*.

[3] Forcillo J., et al. (2016). Morphological and Clinical Findings of Explanted Carpentier-Edwards Perimount Pericardial Valve in the Aortic Position. *J Heart Valve Dis*.

[4] Guo H., Lu C., Huang H. (2017). Long-Term Clinical Outcomes of the Carpentier-Edwards Perimount Pericardial Bioprosthesis in Chinese Patients with Single or Multiple Valve Replacement in Aortic, Mitral, or Tricuspid Positions. *Cardiology*.

[5] Bourguignon T., Lhommet P., El Khoury R. (2016). Very long-term outcomes of the Carpentier-Edwards Perimount aortic valve in patients aged 50-65 years. *European Journal of Cardio-Thoracic Surgery*.

[6] Johnston D. R., Soltesz E. G., Vakil N. (2015). Long-term durability of bioprosthetic aortic valves: implications from 12,569 implants. *The Annals of Thoracic Surgery*.

[7] Bourguignon T., Bouquiaux-Stablo A.-L., Candolfi P. (2015). Very long-term outcomes of the carpentier-edwards perimount valve in aortic position. *The Annals of Thoracic Surgery*.

[8] Totaro P., Degno N., Zaidi A., Youhana A., Argano V. (2005). Carpentier-Edwards PERIMOUNT Magna bioprosthesis: A stented valve with stentless performance?. *The Journal of Thoracic and Cardiovascular Surgery*.

[9] Dalmau M. J., González-Santos J. M., López-Rodríguez J., Bueno M., Arribas A. (2006). The Carpentier-Edwards Perimount Magna aortic xenograft: A new design with an improved hemodynamic performance. *Interactive CardioVascular and Thoracic Surgery*.

[10] Minardi G., Pulignano G., Del Sindaco D. (2011). Early Doppler-echocardiography evaluation of Carpentier-Edwards Standard and Carpentier-Edwards Magna aortic prosthetic valve: Comparison of hemodynamic performance. *Cardiovascular Ultrasound*.

[11] Mizoguchi H., Sakaki M., Inoue K. (2012). Primary echocardiographic results of the Carpentier-Edwards Perimount Magna. *Journal of Medical Ultrasonics*.

[12] Wendt D., Sthle S., Piotrowski J. A. (2012). Comparison of flow dynamics of Perimount Magna and Magna Ease aortic valve prostheses. *Biomedizinische Technik. Biomedical Engineering*.

[13] Wendt D. (2015). The investigation of systolic and diastolic leaflet kinematics of bioprostheses with a new in-vitro test method. *Minim Invasive Ther Allied Technol*.

[14] Wendt D., Stühle S., Hou G. (2011). Development and In Vitro Characterization of a New Artificial Flow Channel. *Artificial Organs*.

[15] Raghav V., Okafor I., Quach M., Dang L., Marquez S., Yoganathan A. P. (2016). Long-Term Durability of Carpentier-Edwards Magna Ease Valve: A One Billion Cycle in Vitro Study. *The Annals of Thoracic Surgery*.

[16] Bach D. S., Patel H. J., Kolias T. J., Deeb M. (2016). Randomized comparison of exercise haemodynamics of Freestyle, Magna Ease and Trifecta bioprostheses after aortic valve replacement for severe aortic stenosis. *European Journal of Cardio-Thoracic Surgery*.

[17] Lim W. L., Chew Y. T., Chew T. C., Low H. T. (2001). Pulsatile flow studies of a porcine bioprosthetic aortic valve in vitro: PIV measurements and shear-induced blood damage. *Journal of Biomechanics*.

[18] Ugur M., Suri R. M., Daly R. C. (2014). Comparison of early hemodynamic performance of 3 aortic valve bioprostheses. *The Journal of Thoracic and Cardiovascular Surgery*.

[19] Bäck M., Gasser T. C., Michel J.-B., Caligiuri G. (2013). Biomechanical factors in the biology of aortic wall and aortic valve diseases. *Cardiovascular Research*.

[20] Wasilewski J., Głowacki J., Poloński L. (2013). Not at random location of atherosclerotic lesions in thoracic aorta and their prognostic significance in relation to the risk of cardiovascular events. *Polish Journal of Radiology*.

[21] Nishimura R. A., Otto C. M., Bonow R. O. (2014). 2014 AHA/ACC guideline for the management of patients with valvular heart disease: a report of the American College of Cardiology/American Heart Association Task Force on Practice Guidelines. *Circulation*.

[22] Carey R. F., Herman B. A. (1989). The effects of a glycerin-based blood analog on the testing of bioprosthetic heart valves. *Journal of Biomechanics*.

